# Effect of Dexamethasone-Loaded PLGA Nanoparticles on Oral Mucositis Induced by 5-Fluorouracil

**DOI:** 10.3390/pharmaceutics13010053

**Published:** 2021-01-04

**Authors:** Susana Barbosa Ribeiro, Aurigena Antunes de Araújo, Maisie Mitchele Barbosa Oliveira, Alaine Maria dos Santos Silva, Arnóbio Antônio da Silva-Júnior, Gerlane Coelho Bernardo Guerra, Gerly Anne de Castro Brito, Renata Ferreira de Carvalho Leitão, Raimundo Fernandes de Araújo Júnior, Vinícius Barreto Garcia, Roseane Carvalho Vasconcelos, Caroline Addison Carvalho Xavier de Medeiros

**Affiliations:** 1 Post Graduate Program Biotechnology-RENORBIO, Federal University of Rio Grande do Norte, 3000 Senador Salgado Filho Ave, Lagoa Nova, Natal RN 59078-970, Brazil; susa05@gmail.com (S.B.R.); maisie.barbosa@gmail.com (M.M.B.O.); 2 Post Graduate Program Dental Sciences, Post Graduate Program Pharmaceutical Science, Department of Biophysics and Pharmacology, Federal University of Rio Grande do Norte, 3000 Senador Salgado Filho Ave, Lagoa Nova, Natal RN 59078-970, Brazil; auriprinino@gmail.com; 3 Laboratory of Pharmaceutical Technology & Biotechnology (TecBioFar), Post Graduate Program Pharmaceutical Sciences, Pharmacy Department, Federal University of Rio Grande do Norte, General Gustavo Cordeiro de Faria St, Petrópolis, Natal RN 59012-570, Brazil; alaine.maria@hotmail.com (A.M.d.S.S.); arnobiosilva@gmail.com (A.A.d.S.-J.); 4 Post Graduate Program Biochemistry and Molecular Biology, Post Graduate Program Pharmaceutical Science, Department of Biophysics and Pharmacology, Federal University of Rio Grande do Norte, 3000 Senador Salgado Filho Ave, Lagoa Nova, Natal RN 59078-970, Brazil; gerlaneguerra@hotmail.com; 5 Post Graduate Program Morphofunctional Sciences, Department of Morphology, Faculty of Medicine, Federal University of Ceará, Delmiro de Farias St, Rodolfo Teófilo, Fortaleza CE 60416-030, Brazil; gerlybrito@gmail.com (G.A.d.C.B.); renata.carvalho@ufc.br (R.F.d.C.L.); 6 Post Graduate Program Functional and Structural Biology, Post Graduate Program Health Science, Department of Morphology, Federal University of Rio Grande do Norte, 3000 Senador Salgado Filho Ave, Lagoa Nova, Natal RN 59078-970, Brazil; araujojr.morfologia@gmail.com; 7 Post Graduate Program Health Science, Federal University of Rio Grande do Norte, General Gustavo Cordeiro de Faria St, Petrópolis, Natal RN 59012-570, Brazil; vbgbiomed@gmail.com; 8 Dentist of the Municipal Health Department Natal-RN, Laranja St, Cidade Nova, Natal RN 59072-570, Brazil; roseane2202@gmail.com; 9 Post Graduate Program Biochemistry and Molecular Biology, Department of Biophysics and Pharmacology, Federal University of Rio Grande do Norte, 3000 Senador Salgado Filho Ave, Lagoa Nova, Natal RN 59078-970, Brazil

**Keywords:** 5-fluorouracil, PLGA, nanoparticles, oral mucositis

## Abstract

Oral mucositis (OM) is characterized by the presence of severe ulcers in the oral region that affects patients treated with chemotherapy. It occurs in almost all patients who receive radiotherapy of the head and neck, as well as patients who undergo hematopoietic cell transplantation. The pathophysiology of OM is complex, and there is no effective therapy. The aim of this study was to evaluate the effect of dexamethasone-loaded poly(d,l-Lactic-*co*-glycolic) nanoparticles (PLGA-DEX NPs) on an OM model induced in hamsters. The NPs were synthesized using the emulsification-solvent evaporation method and were characterized by the size, zeta potential, encapsulation efficiency, atomic force microscopy, physicochemical stability, and the in vitro release. The OM was induced by the administration of 5-FU on the first and second days and mechanical trauma on the 4th day of the experiment. PLGA-DEX NPs were administered to treat OM. The animals were euthanized on the 10th day. Macroscopic and histopathological analyses were performed, measurement of malonaldehyde (MDA) and ELISA was used to determine the levels of IL-1β and TNF-α. Immunoexpressions of NF-κB, COX-2, and TGF-β were determined by immunohistochemistry, and qRT-PCR was used to quantify the gene expression of the GILZ, MKP1, and NF-κB p65. The PLGA-DEX NPs (0.1 mg/kg) significantly reduced macroscopic and histopathological scores, decreased MDA, TNF-α and IL-1β levels, immunostaining for NF-κB, COX-2, TGF-β, and suppressed NF-κB p65 mRNA expression, but increased GILZ and MKP1 expression.

## 1. Introduction

Oral mucositis (OM) is an acute inflammation of the oral cavity resulting from non-surgical antineoplastic therapy [1]. Tissue injury can be induced by the antimetabolite 5-fluorouracil (5-FU) [2], which causes damage to the DNA of cells and induces the generation of reactive oxygen species (ROS). It also activates signal transduction pathways such as nuclear factor kappa beta (NF-κB), a key element in the development of mucositis. NF-κB stimulates the production of the proinflammatory cytokines TNF-α, IL-1β, and IL-6 [1,3,4] and activates other signaling pathways that contribute to tissue damage in the oral cavity [5,6].

OM affects 76–90% of hematopoietic cell transplant patients treated with chemotherapy and/or radiation therapy [7,8]. This damage can also occur as a result of antineoplastic treatment of solid head and neck tumors [9]. Chemo/radiotherapy causes injury and apoptosis of the epithelial cells in the gastrointestinal tract, with ulcer formation and loss of the basal epithelium integrity. This causes painful sensations and compromises the oral nutrition and hydration of the patient. Furthermore, secondary infections with a high risk of sepsis are common, especially in neutropenic individuals [10,11].

Despite its long history and its impact on patients, there are currently no effective treatment options to prevent or treat mucositis associated with chemoradiation therapy for cancer of the head and neck [9,12]. The goals of mucositis management are to prevent or reduce the severity of toxicity and to manage the associated symptoms, which will, in turn, enable the continued delivery of cancer therapy without interruption or dose reduction and improve the overall prognosis [13]. Previous research has shown that drugs with anti-inflammatory action can prevent OM [14,15,16,17]. Recently, it was shown that dexamethasone (DEX) ameliorated OM induced by 5-FU [5]. This drug is an anti-inflammatory glucocorticoid (GC) available for clinical use [18]. However, the use of GC is limited by adverse effects which are directly related to the dose used [19]. These effects include glycemic imbalance, manifestations of latent diabetes mellitus, electrolyte imbalances, hypertension, cataracts, growth suppression in children, loss of muscle mass with consequent muscle weakness, osteoporosis, Cushing’s syndrome and suppression of the hypothalamic-pituitary-adrenal axis [20]. Thus, various nanoformulations of corticosteroids were proposed to overcome those limitations [21]. These nanoparticles could be naked or functionalized with targeting moieties. This allows them to target passively, through the leaky vasculature or actively, linking to the main cells involved in inflammation, including macrophages, endothelial cells, membrane receptors on inflammatory cells, and even anti-inflammatory genes and cytokines [22]. Various biodegradable nanocarrier strategies have been investigated for the treatment of periodontitis, including polylactic-*co*-glycolic acid (PLGA), chitosan, and silica-derived nanoparticles [23,24,25]. According to Brun and colleagues [26]. The agents modulating inflammation in periodontitis seem to be relevant in terms of efficiency. Moreover, poly(lactic-*co*-glycolic acid) or drugs used as their own carrier appear to be the most interesting nanoparticles in terms of biocompatibility.

Poly(d,l-lactic-*co*-glycolic acid) (PLGA) nanoparticles (NPs) is a copolymer of lactic acid and glycolic acid, with biocompatible and biodegradable properties approved for use in humans by the Food and Drug Administration and the European Medicine Agency (EMA) [27]. The incorporation of drugs into PLGA NPs represents a controlled drug release system that has been widely studied. It constitutes a specific and efficient delivery of the drug to the targeted tissue due to several reasons [28,29]. It has been shown the accumulation of nanocarriers in inflamed tissues, due to inflammation-mediated increased vascular permeability [30,31]. In addition, the reduced diameter of these particles allows them to cross biological membranes [32,33].

Therefore, the encapsulation of dexamethasone on PLGA is an efficient strategy for reducing the required dosage, minimizing eventual side effects. This technological innovation can maintain the therapeutic effects of DEX at lower doses, allowing the use of GC with greater safety. Thus, this study aimed to evaluate the effect of DEX, incorporated into the polymeric NPs of PLGA, on 5-FU-induced experimental OM in golden Syrian hamsters.

## 2. Materials and Methods

### 2.1. Reagents for PLGA Nps

Nanoparticles were formed from a copolymer of PLGA (Sigma-Aldrich, Saint Louis, MO, USA) and polyvinyl alcohol (PVA), with a viscometric molecular mass of 4.7 × 104 g/mol (Vetec, São Paulo, Brazil), and contained DEX disodium phosphate (Ache, Guarulhos, Brazil). Organic solvents were dichloromethane (dielectric constant ε 9.1) and acetone (ε 20.6) (Labsynth, Diadema, Brazil). Purified water (1.3 μS) was prepared from the reverse osmosis purification equipment model OS50 LX (Gehaka, São Paulo, Brazil). All chemicals and reagents used for the synthesis of PLGA NPs were of analytical grade.

### 2.2. Preparation of PLGA NPs Loaded with DEX (PLGA-DEX NPs)

The NPs were prepared by the solvent emulsification-evaporation method, according to a previously standardized nanoparticulate system, which consists of an organic and aqueous phase [34]. The aqueous phase (14 mL), containing the surfactant 0.5% *w/v* PVA in water, was filtered through 0.45 μm membranes [35]. The organic phase (6 mL) contained PLGA 0.5% *w/v*, and a 25:75 *v/v* ratio of dichloromethane and acetone was injected into the aqueous phase, with a burette, at 1 mL/min under magnetic stirring at 720 rpm and 25 °C. The emulsification was carried out in Ultra-Turrax equipment (IKA Labortechnik, Staufen, Germany) with a stirring speed of 20,000 rpm for 18 min. The organic solvent was evaporated at 25 °C under magnetic stirring at 720 rpm overnight [35]. Different quantities of DEX were dissolved with the polymer in the organic phase to provide drug/copolymer ratios of 1:20, 1:10, and 1:2.5. The obtained samples were transferred to hermetically sealed glass vials and stored at 5 °C. All experiments were performed in triplicate, and the data are expressed as mean ± standard deviation (SD).

### 2.3. Particle Size and Zeta Potential Measurements

Mean particle size and polydispersity index (PdI) were assessed by using the cumulative method of analysis, according to the intensity of the dynamic light scattered (DLS) in a particle size analyzer, Zetasizer Nano ZS (Malvern Panalytical, Malvern, UK). Data were collected at 659 nm wavelength, 90° detection angle, and at 25 °C. The correlation worked in parallel mode, and data analyzed by using Zeta Plus^®^ particle sizing version 3.95 software (Malvern Panalytical, Malvern, UK). Zeta potential (ζ potential) measurements were performed in the same equipment applying a field strength applied about 5.9 V·cm^−1^, with PALS zeta potential analyzer software (Malvern Panalytical, Malvern, UK), by using the electrophoretic mobility according to the Helmholtz-Smoluchowski equation. The measurements of at least ten determinations for each sample diluted at 1:100 (*v*/*v*) with purified water were carried out in triplicate, and data expressed as mean ± standard deviation (SD) [34].

### 2.4. Drug-Loading Efficiency of PLGA NPs with DEX

The PLGA-DEX NP samples were centrifuged at 16.0× *g* for 60 min at 4 °C (Eppendorf^®^ Microcentrifuge 5404R), using an ultra-centrifugal filter (Vivaspin 2, Ultra-15 MWCO 10 kDa, Sartorius, Gottingen, Germany). The drug present in the supernatant was analyzed by UV-vis spectrophotometry, previously validated at 243 nm (the maximum absorption wavelength for DEX). Encapsulation efficiency (EE%) and drug-loading (DL%) were calculated using Equations (1) and (2), respectively. All analyses were performed in triplicate, and the data are expressed as mean ± SD [35].

(1) $$EE\;\left(\%\right)=\frac{\left(total\;drug-drug\;in\;supernatant\right)}{total\;drug}\;\times 100$$

(2) $$DL\;\left(\%\right)=\frac{total\;drug\;in\;nanoparticle}{total\;nanopaticles}\times 100$$

### 2.5. Atomic Force Microscopy (AFM)

The shape and surface of PLGA NPs, with and without DEX, were observed using atomic force microscopy (AFM) (Shimadzu, Tokyo, Japan). The dispersions were diluted just before analysis, with purified water in a proportion of 1:50 (*v*/*v*), and transferred to a coverslip, which was placed after the addition of the nanoparticles in a desiccator for 24 h. Samples were then analyzed using an SPM-9700 (Shimadzu, Tokyo, Japan) AFM microscope at a temperature of 25 °C with a 1 Hz scan rate [34].

### 2.6. Physicochemical Stability

DEX-loaded formulations and drug-free formulations were stored in hermetically sealed bottles at 5 °C for 5 weeks. Every 7 days, samples were collected to determine the particle size and zeta potential. Measurements were performed at 25 °C. All analyses were performed in triplicate, and the data are expressed as mean ± SD [34].

### 2.7. In Vitro Drug Release

For the in vitro release study of DEX, Franz vertical diffusion static cells (Crown Scientific, Somerville, MA, USA) thermostated at 37 ± 0.5 °C were used. In the donor compartment, 2 mL of different colloidal dispersions were added. This remained hermetically sealed and separated from the recipient compartment by a synthetic 0.45 μm cellulose acetate filter, previously hydrated in phosphate buffer for 24 h. The receiver compartment was filled with 11 mL of phosphate buffer solution, adjusted to pH 7.4, and magnetically stirred at 360 rpm throughout the experiment. At specific intervals, 1 mL aliquots were collected, and the drug was analyzed at 243 nm, which was maximum absorption wavelength UV-vis spectrophotometry. The standard curve for DEX analyses was constructed using the same used phosphate buffer solution at the experiments, using cell cuvette of 1 cm, at 25 °C, and previously validated. The same volume of buffer solution was added to maintain sink conditions. The drug release data were analyzed using linear regression, according to mathematical models, to determine the DEX release mechanism present in PLGA NPs. The correlation coefficient [R^2^] was determined in each case, and consequently, the release orders were determined. All analyses were performed in triplicate, and the data are expressed as mean ± SD [34].

### 2.8. Induction of OM by 5-FU and Experimental Groups of Hamsters

This research was approved by the Ethics Committee on the Use of Animals of the Federal University of Rio Grande do Norte (CEUA-UFRN), permit number 002001/2017. The experimental OM model was induced in golden Syrian hamsters (*Mesocricetus auratus*) males weighing 180 g. They were maintained with rations, water ad libitum, at a temperature of 22 ± 2 °C, and a light/dark cycle of 12 h [15,36]. OM was induced by two intraperitoneal (i.p.) injections of 5-FU (Fauldfluor^®^, Libbs pharmaceutical Ltd.a, São Paulo, Brazil) at doses of 60 mg/kg and 40 mg/kg, on the first and second day, respectively, followed by mechanical trauma (MT) on day 4, based on a previously described experimental oral mucositis model [37]. The mechanical trauma was performed under anesthesia [2% xylazine hydrochloride (10 mg/kg, i.p.; Xilazin, Syntec of Brazil Ltd.a, São Paulo, Brazil) and 10% ketamine hydrochloride (80 mg/kg, i.p.; Cetamin, Syntec of Brazil Ltd.a, São Paulo, Brazil)] using a sterile 25 × 7 mm needle to superficially scratch the mucosa of the right cheek pouch to potentiate oral mucositis. The hamsters were euthanized with 2% thiopental (100 mg/kg, i.p.) (Thiopentax, CRISTÁLIA- Pharmaceutical Chemicals Ltd.a, São Paulo, Brazil) on the 10th day of the experimental model [5].

The treated groups were divided into 3 subgroups that differ only in the concentration of dexamethasone (DEX) encapsulated in PLGA NPs (0.1, 0.5, or 1 mg/kg). These groups (PLGA-DEX) were subjected to OM by 5-FU and mechanical trauma [38] and received the administration of PLGA-DEX NPs (0.1, 0.5, or 1 mg/kg; i.p.) once a day, for 10 days, 60 min before the 5-FU administration, on the 1st and second days. The control animals were divided into 3 control subgroups: a group of healthy hamsters, not subjected to OM, that received no treatment (normal); a group of animals that received only mechanical trauma and daily i.p. injections of 0.4 mL of purified water (trauma) and hamsters that received 5-FU, mechanical trauma and daily i.p. injections of purified water (5-FU).

The animals were euthanized on the 10th day of the experimental model, and the cheek pouches were photographed, subjected to macroscopic analysis and harvested for the following analyses: histopathological, immunohistochemistry, cytokines, measurement of malonaldehyde (MDA), and real-time quantitative polymerase chain reaction (qRT-PCR) considering at least five samples per group.

### 2.9. Macroscopic and Histopathological Analysis of Oral Mucosa

On the 10th day, the oral mucosa was exposed and evaluated in a single-blinded fashion and graded as follows: Score 0: completely healthy mucosa, without erosion or vasodilation. Score 1: the presence of erythema, with no evidence of mucosal erosion. Score 2: severe erythema, vasodilation, and superficial erosion. Score 3: formation of ulcers on one or more faces, affecting no more than 25% of the mucosal surface area, severe erythema, and vasodilation. Score 4: cumulative ulcer formation, reaching approximately 50% of the mucosal surface area. Score 5: complete ulceration, making it impossible to expose the mucosa [16,39].

For histopathological analysis, the oral mucosa was fixed in a 10% formaldehyde buffered solution. The paraffin blocks with tissue were cut into 5 μm-thick sections for hematoxylin–eosin staining (H&E) and examined by light microscopy 40× (Nikon E200 LED, Nikon Corporation, Tokyo, Japan) and then scanned with Pannoramic MIDI II scanner (3DHISTECH Ltd., Budapest, Hungary); images were obtained using the Pannoramic Viewer software (3DHISTECH Ltd., Budapest, Hungary). Each specimen was classified into scores. Score 1: normal epithelium, connective tissue without vasodilation, absence or slight cellular infiltration, absence of hemorrhagic areas, ulcerations, or abscesses. Score 2: areas with mild vasodilation or reepithelialization, mild inflammatory infiltration with mononuclear prevalence, absence of hemorrhagic areas, edema, ulcerations, or abscesses. Score 3: moderate vasodilation, areas of epithelial degeneration, inflammatory infiltration with the prevalence of neutrophils, presence of hemorrhagic areas, edema and eventual ulceration, and absence of abscesses. Score 4: severe vasodilation and inflammatory infiltrate with the presence of neutrophils [40,41].

### 2.10. Determination of Cytokine and Malonaldehyde Levels

Cytokine quantification was developed using the enzyme-linked immunosorbent assay (ELISA) with an R&D Systems kit (Minneapolis, MN, USA) [42]. Initially, the primary antibodies were incubated in Nunc-type microplates for 16 h at 4 °C. The next day, the biological samples were homogenized with phosphate buffer. The plates incubated the previous day were washed with tween-20 to block the wells of these plates with bovine serum albumin. After resting and washing, the plates were incubated with the samples for 2 h at 4 °C. Then, tween-20 was used; the detection antibodies were added for tumor necrosis factor-alpha (TNF-α) (detection range: 62.5–4000 pg/mL; sensitivity: 50 ng/mL of recombinant rat TNF-α), and interleukin 1 beta (range of detection: 62.5 to 4000 pg/mL; sensitivity: 12.5 ng/mL of recombinant rat IL-1β) and the plates remained at rest under the same conditions as in the previous step. Streptavidin (tetramethylbenzidine and hydrogen peroxide) was then added to the plate wells, followed by the stop solution. Finally, the plates were read at 490 nm using an ELISA plate reader Polaris (Celer, Belo Horizonte, Brazil) [5].

The MDA content was quantified [14]. Samples of the oral mucosa were homogenized with Tris-HCl buffer 1:5 (*w*/*v*) at 4 °C and centrifuged at 2500× *g* and 4 °C for 10 min. Homogenate supernatants were used to determine the concentration of MDA. The absorbance of each sample was measured at 586 nm. The results are expressed in nanomoles of MDA per gram of tissue.

### 2.11. Immunohistochemical Analysis

Immunohistochemistry was developed using the standardized method of streptavidin-biotin-peroxidase. Thin sections of mucosal tissue (3 μm) were obtained with a microtome and transferred to silanized slides Star Frost Advanced Adhesive (Knittel, Braunschweig, Germany) that were dewaxed and rehydrated with subsequent antigenic recovery by proteinase K. To block endogenous peroxidase, hydrogen peroxide was used, and sections were incubated with primary antibodies (Santa Cruz Biotechnology, INTERPRISE, Paulínia, Brazil) for COX2 (1:400), TGFβ (1:400), and NF-kβ (1:400); after 18 h at 4 °C, the excess of the primary antibody was removed. The secondary antibody was added (Biocare Medical, Concord, CA, USA) at 25 °C, followed by the horseradish peroxidase conjugate (Biocare Medical, Concord, CA, USA). Immunoreactivity to the various proteins was visualized with a colorimetric-based detection kit following the protocol provided by the manufacturer (TrekAvidin-HRP Label, Biocare Medical, Concord, CA, USA) [5]. Negative control sections were simultaneously processed as described above, but the primary antibody was replaced with antibody diluent, and none showed COX2, TGFβ or NF-kβ immunoreactivity.

The specimens were evaluated by optical planimetry microscopy (Nikon E200 LED, Nikon Corporation, Tokyo, Japan) with a high-power objective lens 40× [5]. The intensity of the immunostaining was categorized as mild or intense by two examiners in a double-blind mode and classified into scores. Score 1: the absence of positive cells (0%). Score 2: a small number of positive cells or isolated cells (<10%). Score 3: moderate number of positive cells (11–50%). Score 4: a large number of positive cells (>50%) [5,41].

### 2.12. Real-Time Quantitative Polymerase Chain Reaction

In the qRT-PCR, the homogenate was prepared from oral mucosa samples using the Trizol reagent (Life Technologies, Carlsbad, CA, USA) to extract nucleic acids. RNA was isolated from DNA using the SV total RNA isolation system kit (Promega, Madison, WI, USA), and NanoDrop equipment (Thermo Scientific NanoDrop Products, Wilmington, DE, USA) was used to determine the level and purity of the RNA present in the extracted volume (Desjardins and Conklin, 2010). The mRNA was converted into complementary deoxyribonucleic acid (cDNA) using reverse transcriptase (high-capacity cDNA reverse transcription kit, Foster City, CA, USA) and the following thermal cycle schedule: 10 min at 25 °C; 120 min at 37 °C; 5 min at 85 °C and ∞ 4 °C. A final volume of 20 µL of cDNA was obtained. Primer Express^TM^ software version 3.0.1 (Applied Biosystems, Foster City, CA, USA) was used to design the primers used in this analysis (Table 1).

The reaction for quantification of mRNA expression in real time was performed in a 96-well plate using the reverse (R) and forward (F) primers, cDNA, 5 μL Power up SYBR Green Master Mix, free nuclease water, and 2 μL cDNA. The run was developed on the Step One Plus^TM^ Real-Time PCR system thermocycler (Applied Biosystems, Foster City, CA, USA) following the temperature cycle of 95 °C for 20 s, followed by 40 cycles of 3 s at 95 °C, and 40 cycles of 30 s at 58 °C. The specificity of the PCR products was confirmed by the melting curves. The comparative cycle threshold (Ct) was applied to determine the expression of the genes, with Ct being the number of cycles necessary to observe the first fluorescence signal that exceeds the threshold (baseline), representing the beginning of the exponential amplification of the genetic material. This method analyzes the gene expression of the sample in the control (normal group) using the Ct values. The expression data were standardized using the reference gene GADPH in the formula 2^−ΔΔCt^ [43,44].

## 3. Results

### 3.1. Determination of Particle Size, Zeta Potential, and Encapsulation Efficiency of PLGA-DEX NPs

The NPs were prepared by the emulsification-solvent evaporation method, using a concentration of 0.5% PLGA and 0.5% PVA to the solvent dichloromethane: acetone 25:75 (*v*/*v*). Drug-free NPs and NPs with different drug and copolymer ratios (DEX: PLGA 1:20, 1:10, and 1:2.5) were prepared.

The results showed the formation of small particles (207.1 ± 1.0 nm to 317.2 ± 4.7 nm), with desirable values of zeta potential (−2.3 ± 2.1 mV to −19.3 ± 0.2 mV) and PDI (0.190 ± 0.39 nm to 0.394 ± 0.53 nm). The highest drug encapsulation efficiency was approximately 65%, observed for formulations containing a 1:10 and 1:2.5 DEX: PLGA weight ratio. These formulations resulted in a drug-loaded of approximately 0.67% and 0.87%, respectively, which corresponds to a final drug concentration of 250 µg/mL and 1 mg/mL, respectively (Table 2).

### 3.2. Atomic Force Microscopy

**Figure 1:**
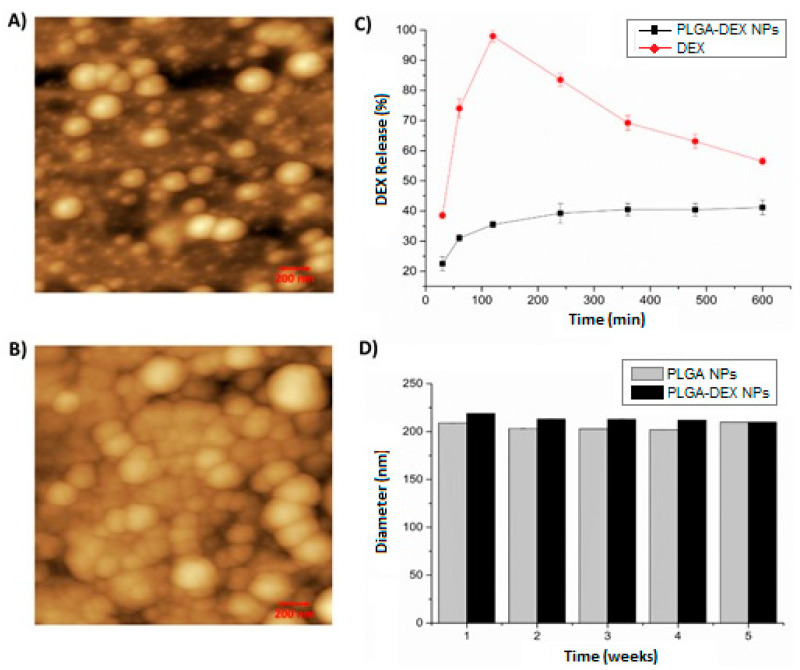
Atomic force microscopy (AFM)—2D images—200 nm scale. (**A**) The nanoparticle of poly(d, Lactic-*co*-glycolic) nanoparticles (PLGA NP). (**B**) dexamethasone-loaded poly (d, Lactic-*co*-glycolic) nanoparticles (PLGA-DEX NP). (**C**) In vitro release profile of dexamethasone free in solution (DEX) and PLGA-DEX NP. (**D**) Physical stability of PLGA NP and PLGA-DEX NP for 5 weeks.

AFM images (Figure 1) show the morphological aspects of the shape and surface of the particles. In the 2D images, drug-free PLGA NPs (Figure 1A) and DEX-loaded PLGA NPs (Figure 1B) were observed. The addition of DEX to PLGA NPs did not change the shape of the NPs, which remained spherical.

According to the in vitro release profile, DEX in PLGA NPs presented a release rate of approximately 42% (PLGA-DEX NPs), featuring a slow, sustainable, prolonged-release profile with the capacity to release DEX for up to 600 min. In contrast, the release of free (isolated) DEX showed a release rate close to 100% in just 120 min, followed by decay, demonstrating a rapid release profile (Figure 1C).

The diameter of PLGA NPs without DEX remained below 220 nm. The size of the NPs containing DEX for all systems was approximately 220 nm, which is close to the values found by DLS (Figure 1D).

### 3.3. Macroscopic and Histopathological Analysis of OM

**Figure 2:**
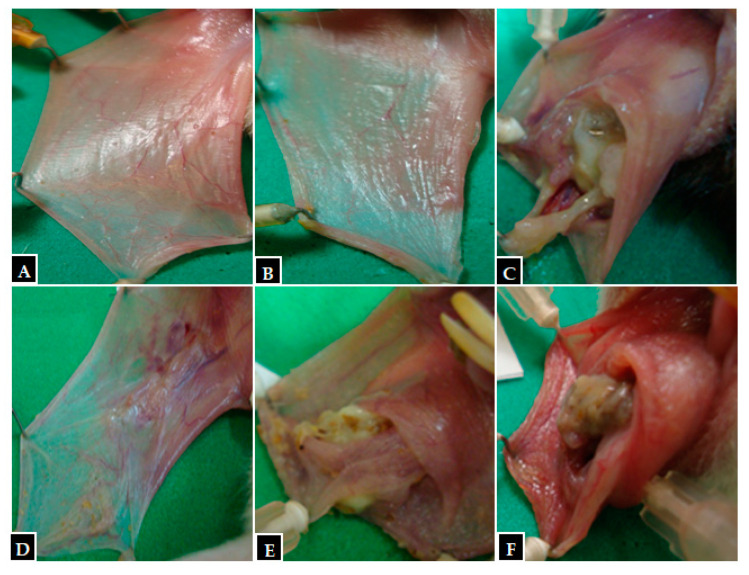
Macroscopic images from hamster oral mucosa with oral mucositis (OM) induced by 5-fluorouracil (5-FU) and mechanical trauma (MT), treated with PLGA-DEX NPs. (**A**) Normal group: animals not subjected to induction of OM received daily administrations of purified water i.p. (**B**) Trauma group: animals subjected to MT only, without OM inductions, received purified water i.p. daily. (**C**) Group 5-FU: animals with OM induced by 5-FU and MT received purified water i.p. daily. PLGA-DEX NP groups (**D**) 0.1 mg/kg, (**E**) 0.5 mg/kg, (**F**) 1 mg/kg: animals with OM treated with daily administrations of PLGA-DEX NPs i.p. at the dose specified for each of the three groups.

**Figure 3:**
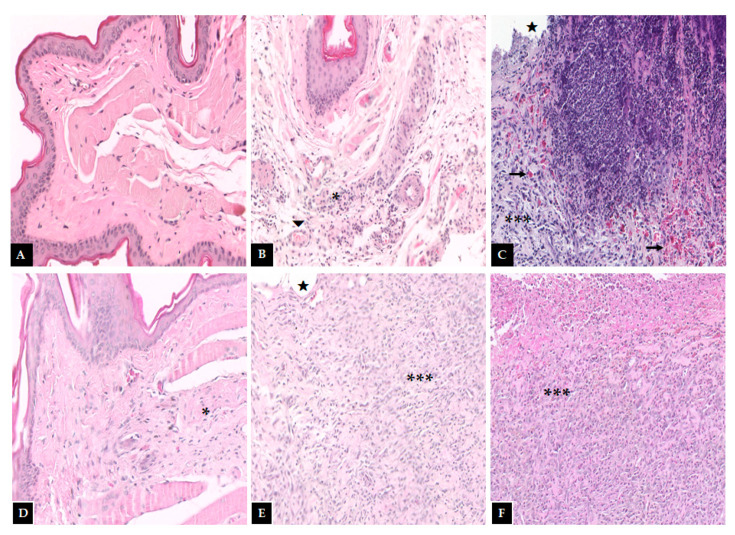
Histopathological examination of the hamster oral mucosa. OM was induced by 5-FU and MT, treated with PLGA-DEX NPs. (**A**) Normal group: animals without histopathological changes in oral mucosa. (**B**) Trauma group: animals subjected to the induction of excoriations in the oral mucosa, without OM; there are blood vessels (triangle) and a small region of inflammatory infiltrate (an asterisk) dispersed in the conjunctive. (**C**) Group 5-FU: animals with untreated OM, characterized by the presence of ulcers (star), intense inflammatory infiltrate (three asterisks), and hemorrhagic foci (arrow) dispersed throughout the region. PLGA-DEX groups (**D**) 0.1 mg/kg, (**E**) 0.5 mg/kg, (**F**) 1 mg/kg: animals with OM treated with DEX-loaded PLGA NPs. The groups treated with PLGA-DEX NPs 0.5 and 1 mg/kg presented intense inflammatory infiltrate (three asterisk) and ulcers (star). The group treated with PLGA-DEX NPs 0.1 mg/kg presented a reduction in inflammation, marked by a decrease in inflammatory infiltrate (an asterisk).

Macroscopic and histopathological analyses of the oral mucosa of the normal group (score 0—Figure 2, Table 3); (score 1—Figure 3, Table 4) showed absence of erosion, vasodilation, ulcerations, or abscesses in the connective tissue, or characteristics compatible with healthy mucosa. In the trauma group (score 1—Figure 2, Table 3); (score 2—Figure 3, Table 4), areas with mild vasodilation, mild inflammatory infiltration, and absence of ulcers were observed. The statistical analysis showed differences between the normal and trauma animals compared to the 5-FU-treated group (Table 3 and Table 4). The 5-FU-treated animals (score 4—Figure 2, Table 3) presented cumulative ulcer formation, making it impossible to expose the mucosa for macroscopic analysis. While in the histopathological aspect (score 4—Figure 3, Table 4), they showed severe vasodilation, intense inflammatory infiltrates, abscesses, and ulcers. The group treated with PLGA-DEX NPs 0.1 mg/kg (score 1—Figure 2, Table 3); (score 2—Figure 3, Table 4) displayed areas with slight vasodilation or reepithelization, mild inflammatory infiltration, and absence of edema, bleeding, and ulcerations. Animals administered doses of 0.5 or 1 mg/kg of PLGA-DEX NPs (Figure 2 and Figure 3, Table 3 and Table 4) presented epithelium similar to those of 5-FU-treated animals.

### 3.4. Determination of Cytokine and Malonaldehyde Levels

**Figure 4:**
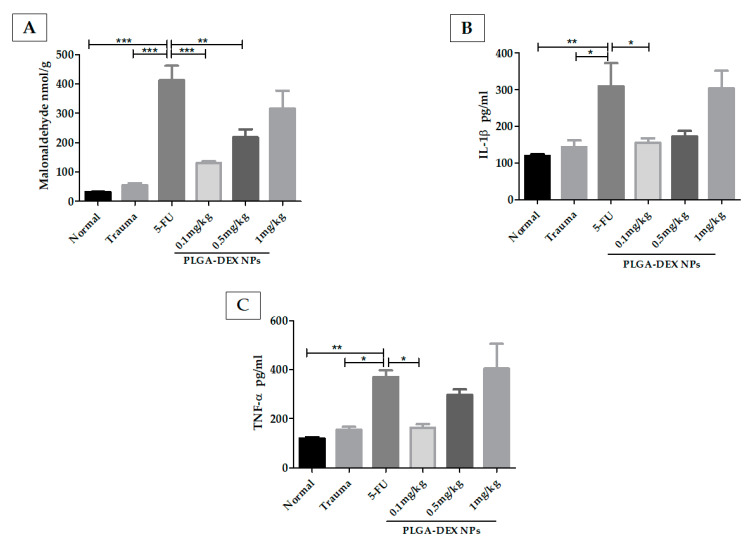
Malonaldehyde (MDA) and cytokine quantifications in the hamster oral mucosa to evaluate the therapeutic outcome of PLGA-DEX NPs in OM induced by 5-FU. (**A**) MDA. (**B**) IL-1β. (**C**) TNF-α. Normal group: animals with healthy oral mucosa. Trauma group: animals subjected only to excoriations in the oral mucosa, but they do not present OM. Group 5-FU: animals with untreated OM only received purified water, i.p. The PLGA-DEX NPs groups (0.1; 0.5 or 1 mg/kg) received 5-FU and were subjected to MT with consequent OM induction, which was treated with PLGA-DEX NP i.p. in the dose corresponding to the group to which they belonged. The results are presented as the mean ± standard error (*n* = 5). * *p* < 0.05, ** *p* < 0.01, *** *p* < 0.001 (ANOVA with Tukey’s posttest).

MDA (Figure 4A) was elevated in the 5-FU group, compared to the normal and trauma groups. Comparison of the 5-FU group with the PLGA-DEX NPs 0.1 mg/kg and 0.5 mg/kg group showed a statistical difference, (*** *p* < 0.001; ** *p* < 0.01).

The concentration of IL-1β (Figure 4B) was higher in the 5-FU group than in the groups without OM (normal and trauma groups). The animals treated with PLGA-DEX NPs, 0.1 mg/kg, showed a significant reduction in IL-1β. TNF-α (Figure 4C) was at similar levels in the normal, trauma, and PLGA-DEX 0.1 mg/kg groups, showing statistically lower values than the 5-FU group (** *p* < 0.01; * *p* < 0.05).

### 3.5. Immunohistochemistry for NF-κB, TGFβ, and COX-2

**Figure 5:**
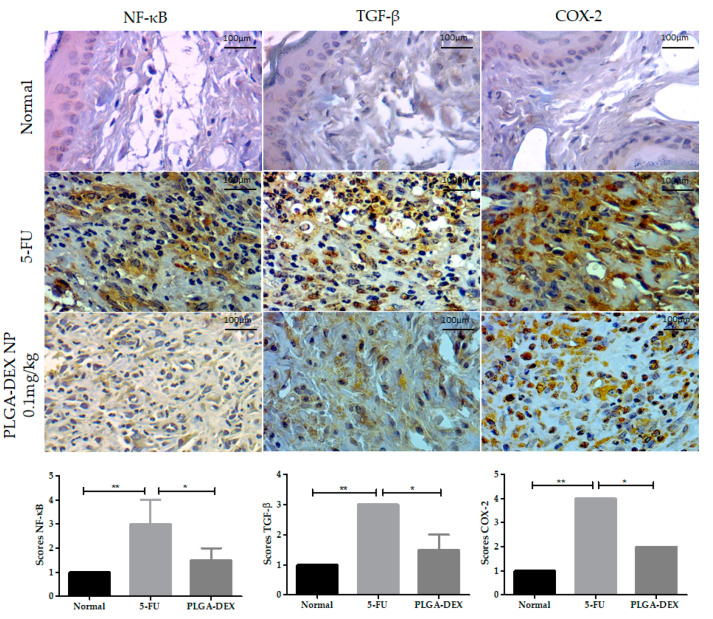
Immunoexpression photomicrographs of NF-κB, TGF-β, and COX-2 in the normal, 5-FU, and PLGA-DEX 0.1 mg/kg groups. The immunostaining of these proteins was greater in the 5-FU group, while the normal and PLGA-DEX NPs groups exhibited low expression, bars indicating 100 µm. Expression scores for NF-κB, TGF-β, and COX-2 with a 95% confidence interval. * *p* < 0.05; ** *p* < 0.01 (Kruskal–Wallis test, followed by Dunn’s test for post hoc comparisons).

The 5-FU group showed higher immunostaining (score 3) for nuclear factor kappa B (NF-κB) than the normal animals (score 1) and the group treated with PLGA-DEX 0.1 mg/kg (score 1.5), whose reduction in immunostaining was observed (* *p* < 0.05 Vs. 5-FU group). The 5-FU animals showed more intense labeling for transforming growth factor-beta (TGF-β) (score 3), while the PLGA-DEX group presented intermediate values (score 1.5) (* *p* < 0.05 vs. 5-FU group), and the lowest score was observed in the normal group (score 1). Statistical differences were found between the groups for the expression of COX-2; the 5-FU group showed the greater intensity of marking (score 4) compared to the groups PLGA-DEX NPs 0.1 mg/kg (score 2) (* *p* < 0.05 vs. 5-FU group) and normal (score 1) (Figure 5).

### 3.6. Quantification of Gene Expression by Real-Time Quantitative Polymerase Chain Reaction for NF-κB, MKP 1, and GILZ

**Figure 6:**
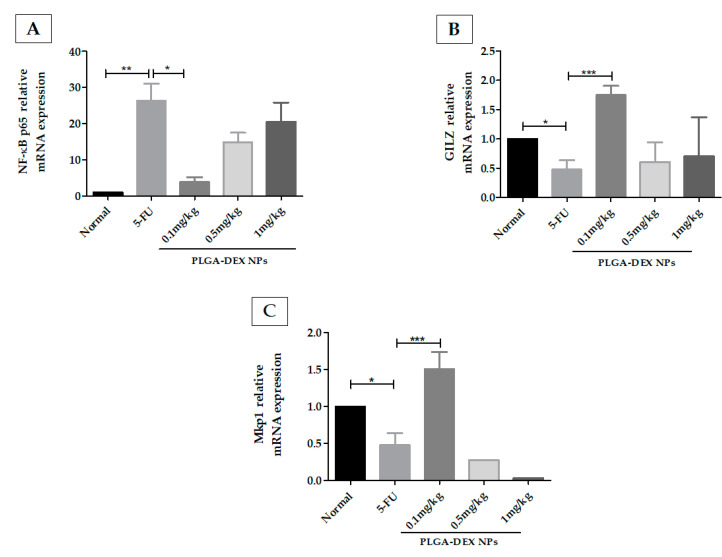
(**A**) q-RT PCR for NF-kB p65, (**B**) glucocorticoid-induced leucine zipper (GILZ), (**C**) MAPK phosphatase 1 (MKP1). 5-FU increased the expression of the NF-κB p65 genes and decreased the gene expression of GILZ and MKP1, compared to the normal group. The animals in the PLGA-DEX group 0.1 mg/kg showed increased GILZ and MKP1 gene expression as well as reduced expression of the NF-κB p65 gene, compared to the 5-FU group (*n* = 5; * *p* < 0.05, ** *p* < 0.01, *** *p* < 0.001) (ANOVA followed by Tukey’s posttest).

Treatment of OM with PLGA-DEX NPs 0.1 mg/kg reduced mRNA expression of NF-κB compared to the 5-FU group, showing an expression close to that of the normal group. PLGA-DEX 0.5 mg/kg or 1 mg/kg treated groups did not show a significant reduction in NF-κB compared to animals that received only 5-FU (Figure 6A). The PLGA-DEX 0.1 mg/kg treated group showed increased gene expression of mitogen-activated protein kinase phosphatase 1 (MKP1) (Figure 6B) and the glucocorticoid-induced leucine zipper (GILZ) (Figure 6C), compared to the 5-FU group. Animals treated with NP at a dose of 0.5 mg/kg or 1 mg/kg did not show an increase in the concentration of MKP1 or GILZ mRNA, compared to the 5-FU group (* *p* < 0.05; ** *p* < 0.01; *** *p* < 0.001).

## 4. Discussion

In this study, 5-FU induced OM in hamsters, as evidenced in macroscopic analyses by the cumulative formation of ulcers and in the histopathological examination, which showed intense vasodilation with intense inflammatory infiltrate, abscesses and ulcers. 5-FU is a chemotherapy drug that alters cell function, interfering with DNA synthesis and, to a lesser extent, inhibiting the formation of RNA. The main cytotoxic activity of 5-FU is induced by the metabolite fluorodeoxyuridine monophosphate, which interacts with the enzyme thymidylate synthase to block the formation of thymidine triphosphate; this impairs cell growth since thymidine triphosphate is a precursor for DNA molecular synthesis [44]. The treatment with PLGA-DEX 0.1 mg/kg prevented the inflammatory alterations at day 10 when compared with the non-treated group subjected to OM, the 5-FU control group. It should be noted that PLGA-DEX 0.1 mg/kg prevented injuries induced by 5-FU and did not improve healing since the 10th day of the experiment corresponds to maximum mucositis in hamsters [12,16].

In a previous study, we showed that free (isolated) DEX, at a dose of 1 mg/kg, had an anti-inflammatory and protective effect on OM induced by 5-FU in hamsters [44]. In the present study, DEX (0.1 mg/kg) encapsulated in PLGA NPs also reduced inflammatory alterations induced by chemotherapy. The oral mucous membranes of these animals treated with PLGA-DEX NPs 0.1 mg/kg showed areas with slight vasodilation, reepithelialization, mild inflammatory infiltration, absence of edema, hemorrhages, and ulcerations. Therefore, these animals demonstrated a significant reduction in mucosal damage compared to the untreated animals (5-FU group). We observed better therapeutic efficacy in animals treated with PLGA-DEX NPs 0.1 mg/kg, which is a concentration 10 times lower than that used in the work of Ribeiro et al. (2017). DEX is a potent anti-inflammatory agent; however, the occurrence of numerous adverse effects related to the dose and duration of therapy can limit the beneficial results for the patient [45]. Therefore, the incorporation of DEX into PLGA NPs, as demonstrated in this study, has the advantage of allowing therapeutic optimization with lower doses of the glucocorticoid.

The in vitro release profile of free (isolated) DEX demonstrated percentage release of close to 100% in the first 120 min after the start of the test; however, after encapsulation in PLGA NPs, the release rate became slower, remaining controlled for at least 600 min. In a study on functional polymeric NPs for modified DEX release, the release rate of the systems ranged between 2% and 25% [46]. This characteristic suggests that copolymers such as PLGA are suitable for adapting the release of hydrophobic drugs trapped in the polymeric matrix [47]. Corroborating our results, the authors demonstrated that PLGA NPs, loaded with the test substance docetaxel, provide a controlled release profile of the drug since the PLGA polymer needs to be degraded for the diffusion of docetaxel from the NP matrix into the biological environment [48].

In our study, the emulsification solvent evaporation method used for the synthesis of PLGA-DEX NPs gave rise to small particles with desirable zeta potential values. The best results were obtained with diameter particles smaller than 220 nm, which is ideal for cell adsorption [49]. A study on DEX-loaded PLGA NPs used in a human placental model in vitro showed particle sizes of 298 nm, which were larger than those in our study [50]. Previous work has shown that PLGA NPs with a diameter between 100 and 500 nm, can be used to target drugs to specific targets, such as tumors [48,51,52]. NPs with sizes less than 500 nm are hardly phagocytized, while those with sizes greater than 100 nm hinder immune sensitization mechanisms [48].

The zeta potential reflects the surface charge of the particles. PLGA, used as the main polymer dispersed in the organic phase, gave us a negative zeta potential in all our results. This can be explained by considering the nonionic nature of the polymer [53]. The highest drug-loading efficiency was observed in the formulation containing DEX:PLGA in the 1:10 and 1:2.5 weight ratios, with an efficiency of around 65%. A study on the anti-inflammatory activity of poly (lactic acid) (PLA) NPs containing DEX showed an encapsulation efficiency of 66%, a value like that presented in our study [54]. In another study on the encapsulation of DEX in PLGA NPs, the authors used a polymer composition similar to that of our system and the same ratio of organic solvents in the synthetic process. However, the result of the encapsulation efficiency was only 3%, with a final DEX concentration of 125 µg/mL. The encapsulation efficiency of our system was much higher, close to 65%, with DEX concentrations of 250 µg/mL and 1 mg/mL. This was better than that reported by Gómez-Gete et al. (2007), proving the effectiveness of the synthetic method developed in our research [55]. The comparison of drug-free PLGA NPs images with DEX-loaded PLGA NPs confirmed that the drug load did not affect the shape of the NPs, which remained spherical with a smooth surface. It was previously reported that spherical particles with a uniform size distribution show improved drug release kinetics [56,57].

The use of nanoparticulate therapeutic systems can improve the effectiveness of treatments. Since drug-NP conjugates have a diameter between 50 and 800 nm, these fail to pass through the vessels of healthy regions of the body, which have a space between the cells of 15–30 nm [44]. On the contrary, the retention of nanoparticulate formulations becomes favorable in inflamed tissues since there is edema, and consequently, greater space between cells, increasing the probability of localized anti-inflammatory effects [30,31]. The preferential accumulation of nanoparticles by specific sites is explained by the effect of retention and permeability (EPR) [58]. In this study, the best results were found in animals treated with PLGA-DEX NPs 0.1 mg/kg, compared to the higher doses (PLGA-DEX NPs 0.5 mg/kg or PLGA-DEX NPs 1 mg/kg). Larger doses of the drug associated with the PLGA nanoparticle formulation requires an administration of a higher amount of formulation in a short volume of a colloidal dispersion to supply larger doses of the drug. Thus, the accumulation of hydrophobic nanoparticles in a specific site of administration could be expected, reducing the drug diffusion and, consequently, its bioavailability [28,59]. These concepts can explain the results found in the present study, which is not necessarily dose-dependent.

The 5-FU induced the formation of ROS, which activates the nuclear factor kappa beta (NF-κB) signaling pathway that is an essential element for the pathophysiology of OM. NF-κB positively regulates the expression of TNF-α and IL-1β, proinflammatory cytokines involved in the amplification of mucositis signals. In addition, NF-κB promotes activation of COX-2 and TGF-β pathways [59,60].

Our data show that PLGA-DEX NPs 0.1 mg/kg prevented the lesions in the oral mucosa induced by 5-FU, reinforced by a significant reduction of the inflammatory markers involved in its pathophysiology, such as NF-κB, COX-2, TGF-β, proinflammatory cytokines (IL-1β, TNF-α), and MDA. In addition, it was observed a significant increase in the expression of the GILZ gene in the PLGA-DEX NPs 0.1 mg/kg group, compared to the 5-FU group. GILZ is considered a resolution marker of the inflammatory process because it inhibits NF-κB [31]. Corroborating with our findings, it was demonstrated that the anti-inflammatory activity of DEX is associated with inhibition of COX-2 and NF-κB expression [18]. Srinivasan and colleagues have shown that GC enhances GILZ expression [31]. DEX interacts with the glucocorticoid receptor, which is expressed in the cell cytoplasm [61]. The drug–receptor complex migrates to the cell nucleus to bind to the glucocorticoid response elements present in the promoter region of target genes, increasing or suppressing gene expression [62].

The experimental data show reduced mRNA expression of MKP1 in the 5-FU group. The animals treated with PLGA-DEX NPs 0.1 mg/kg showed increased MKP1 mRNA expression. MKP1 inhibits TNF-α, an essential factor involved in the amplification of the oral mucosa damage induced by 5-FU [63,64]. TNF-α has a direct impact on mucosal cells and plays an indirect role in activating signaling pathways that reinforce OM signals, leading to tissue damage, including ceramide, caspase, and NF-kB pathway amplification [65,66]. Thus, the increase in MKP1 and GILZ gene expression by DEX resulted in therapeutic benefits since it blocked the main proinflammatory cytokine signaling pathway involved in the pathophysiology of OM [44].

In the present investigation, we demonstrated that DEX-loaded PLGA nanoparticles were able to prevent clinical signs of oral mucositis induced by the 5-FU chemotherapy. Our data suggest that the controlled release of dexamethasone from PLGA nanoparticles is an efficient strategy to reduce the required dosage, as it showed the same effectiveness as a 10-fold lower dose of free dexamethasone.

## Figures and Tables

**Table 1:** Primer sequences designed in Primers Express™ for qRT-PCR.

Gene/Species	Forward Sequence	Reverse Sequence
GADPH Mesocricetus auratus	GAC TCA TGA CCA CAG TCC ATG C	AGA GGC AGG GAT GAT GTT CTG
GILZ Rattus norvegicus	CCG GCA ACC CGA ATC A	TGA TAG ACC GCC ACC TCC AT
MKP1 Rattus norvegicus	CCT GTA CCT GGG AGT GCT T	CCC AAG GCG TCG AGC ATA T
NF-κB p65 Mesocricetus auratus	GAA GAA GCG AGA CCT GGA GCA A	GTT GAT GGT GCT GAG GGA TGC T

**Table 2:** Diameter, polydispersity index (PDI), zeta potential (PZ), encapsulation efficiency (EE), and drug-loading (DL) in the analysis of PLGA and PLGA-DEX NPs.

Nanoparticle (NPs)	Diameter (nm) ± SD	PDI ±SD	PZ (mV) ± SD	EE (%) ± SD	DL (%) ± SD
PLGA NPs	207.1 ± 1.0	0.263 ± 0.21	−2.3 ± 2.1	-	-
PLGA-DEX NPs 1:2.5	210.0 ± 4.8	0.394 ± 0.53	−19.3 ± 0.2	64.9 ± 0.6	0.87 ± 0.5
PLGA-DEX NPs 1:10	213.3 ± 3.9	0.190 ± 0.39	−14.8 ± 0.5	64.4 ± 0.4	0.67 ± 0.2
PLGA-DEX NPs 1:20	317.5 ± 4.7	0.345 ± 3.2	−8.3 ± 2.1	10.4 ± 2.6	7.17 ± 3.2

Notes: nm (nanometer), standard deviation (SD), millivolt (mV).

**Table 3:** Macroscopic scores from hamster oral mucosa with OM induced by 5-FU and MT. Scores are represented as medians (*n* = 5). * *p* < 0.05; *** *p* < 0.001, vs. group 5-FU (Kruskal–Wallis test and Dunn’s multiple comparison test).

Experimental Groups	Macroscopic Analysis Scores
Normal	0 (0–0) ***
Trauma	1 (0–2) *
5-FU	4 (4–5)
PLGA-DEX 0.1 mg/kg	1 (1–2) *
PLGA-DEX 0.5 mg/kg	4 (4–5)
PLGA-DEX 1 mg/kg	4 (3–4)

**Table 4:** Histopathological scores from hamster oral mucosa with OM induced by 5-FU and MT. Scores are represented as medians (*n* = 5). * *p* < 0.05; ** *p* < 0.01 vs. group 5-FU (Kruskal–Wallis test and Dunn’s multiple comparison test).

Experimental Groups	Histopathological Analysis Scores
Normal	1 (1–1) **
Trauma	2 (1–2) *
5-FU	4 (4–4)
PLGA-DEX 0.1 mg/kg	2(1–2) *
PLGA-DEX 0.5 mg/kg	3 (2–3)
PLGA-DEX 1 mg/kg	4 (3–4)

## Data Availability

Data sharing not applicable.

## References

[1] Sonis S. (2004). A biological approach to mucositis. J. Support. Oncol..

[2] Barasch A., Peterson D.E. (2003). Risk factors for ulcerative oral mucositis in cancer patients: Unanswered questions. Oral Oncol..

[3] Elting L.S., Keefe D.M., Sonis S.T., Garden A.S., Spijkervet F.K.L., Barasch A., Tishler R.B., Canty T.P., Kudrimoti M.K., Vera-Llonch M. (2008). Patient-reported measurements of oral mucositis in head and neck cancer patients treated with radiotherapy with or without chemotherapy: Demonstration of increased frequency, severity, resistance to palliation, and impact on quality of life. Cancer.

[4] Rastogi M., Dwivedi R., Kazi R. (2011). Oral mucositis in head and neck cancer. Eur. J. Cancer Care.

[5] Ribeiro S.B., De Araújo A.A., De Araújo Júnior R.F., De Castro Brito G.A., Leitão R.C., Barbosa M.M., Garcia V.B., Medeiros A.C., De Medeiros C.A.C.X. (2017). Protective effect of dexamethasone on 5-FU-induced oral mucositis in hamsters. PLoS ONE.

[6] Georgiou M., Patapatiou G., Domoxoudis S., Pistevou-Gompaki K., Papanikolaou A. (2012). Oral Mucositis: Understanding the pathology and management. Hippokratia.

[7] Vitale M.C., Modaffari C., Decembrino N., Zhou F.X., Zecca M., Defabianis P. (2017). Preliminary study in a new protocol for the treatment of oral mucositis in pediatric patients undergoing hematopoietic stem cell transplantation (HSCT) and chemotherapy (CT). Lasers Med. Sci..

[8] Vera-Llonch M., Oster G., Ford C.M., Lu J., Sonis S. (2007). Oral mucositis and outcomes of allogeneic hematopoietic stem-cell transplantation in patients with hematologic malignancies. Support. Care Cancer.

[9] Blakaj A., Bonomi M., Gamez M.E., Blakaj D.M. (2019). Oral mucositis in head and neck cancer: Evidence-based management and review of clinical trial data. Oral Oncol..

[10] Basile D., Di Nardo P., Corvaja C., Garattini S.K., Pelizzari G., Lisanti C., Bortot L., Da Ros L., Bartoletti M., Borghi M. (2019). Mucosal Injury during Anti-Cancer Treatment: From Pathobiology to Bedside. Cancers.

[11] Epstein J.B., Thariat J., Bensadoun R.-J., Barasch A., Murphy B.A., Kolnick L., Popplewell L., Maghami E. (2012). Oral complications of cancer and cancer therapy: From cancer treatment to survivorship. CA Cancer J. Clin..

[12] Sonis S.T. (2010). Oral mucositis. Oral Oncol..

[13] Lalla R.V., Bowen J., Barasch A., Elting L., Epstein J., Keefe D.M., McGuire D.B., Migliorati C., Nicolatou-Galitis O., Dmd D.E.P. (2014). MASCC/ISOO clinical practice guidelines for the management of mucositis secondary to cancer therapy. Cancer.

[14] Araújo A.A.d., Araújo L.d.S., Medeiros C.A.C.X.d., Leitão R.F.d.C., Brito G.A.d.C., Costa D.V.d.S., Guerra G.C.B., Garcia V.B., Lima M.L.d.S., Junior R.F.d.A. (2018). Protective effect of angiotensin II receptor blocker against oxidative stress and inflammation in an oral mucositis experimental model. J. Oral Pathol. Med..

[15] Mafra C., Vasconcelos R.C., de Medeiros C., Leitao R.F.C., Brito G.A.C., Costa D., Guerra G.C.B., de Araujo R.F., Medeiros A.C., de Araujo A.A. (2019). Gliclazide Prevents 5-FU-Induced Oral Mucositis by Reducing Oxidative Stress, Inflammation, and P-Selectin Adhesion Molecules. Front. Physiol..

[16] Barbosa M.M., Araújo A.A.d., Júnior R.F.d.A., Guerra G.C.B., Brito G.A.d.C., Leitão R.C., Ribeiro S.B., Tavares E.d.A., Vasconcelos R.C., Garcia V.B. (2018). Telmisartan Modulates the Oral Mucositis Induced by 5-Fluorouracil in Hamsters. Front. Physiol..

[17] Medeiros C.A.C.X., Leitão R.F.C., MacEdo R.N., Barboza D.R.M.M., Gomes A.S., Nogueira N.A.P., Alencar N.M.N., Ribeiro R.A., Brito G.A.C. (2011). Effect of atorvastatin on 5-fluorouracil-induced experimental oral mucositis. Cancer Chemother. Pharmacol..

[18] Ruijters E.J.B., Haenen G.R.M.M., Weseler A.R., Bast A. (2014). The anti-inflammatory efficacy of dexamethasone is protected by (−)-epicatechin. Pharma Nutr..

[19] Bellavance M.-A., Rivest S. (2014). The HPA—Immune Axis and the Immunomodulatory Actions of Glucocorticoids in the Brain. Front. Immunol..

[20] Van Der Goes M.C., Jacobs J.W., Bijlsma J.W. (2014). The value of glucocorticoid co-therapy in different rheumatic diseases—Positive and adverse effects. Arthritis Res. Ther..

[21] Youshia J., Lamprecht A. (2015). Size-dependent nanoparticulate drug delivery in inflammatory bowel diseases. Expert Opin. Drug Deliv..

[22] Brusini R., Varna M., Couvreur P. (2020). Advanced nanomedicines for the treatment of inflammatory diseases. Adv. Drug Deliv. Rev..

[23] Brun A., Moignot N., Colombier M.L., Dursun E. (2019). Towards the nano-control of periodontal inflammation?. Oral Dis..

[24] Carrouel F., Viennot S., Ottolenghi L., Gaillard C., Bourgeois D. (2020). Nanoparticles as Anti-Microbial, Anti-Inflammatory, and Remineralizing Agents in Oral Care Cosmetics: A Review of the Current Situation. Nanomaterial.

[25] Cafferata E.A., Alvarez C., Diaz K.T., Maureira M., Monasterio G., González F.E., Covarrubias C., Vernal R. (2019). Multifunctional nanocarriers for the treatment of periodontitis: Immunomodulatory, antimicrobial, and regenerative strategies. Oral Dis..

[26] Brun A., Moignot N., Colombier M.-L., Dursun E. (2020). Emerging Nanotechnology in Non-Surgical Periodontal Therapy in Animal Models: A Systematic Review. Nanomaterial.

[27] Wang Z., Liang R., Jiang X., Xie J., Cai P., Chen H., Zhan X., Lei D., Zhao J., Zheng L. (2019). Electrospun PLGA/PCL/OCP nanofiber membranes promote osteogenic differentiation of mesenchymal stem cells (MSCs). Mater. Sci. Eng. C.

[28] Allavena P., Palmioli A., Avigni R., Sironi M., La Ferla B., Maeda A. (2020). PLGA Based Nanoparticles for the Monocyte-Mediated Anti-Tumor Drug Delivery System. J. Biomed. Nanotechnol..

[29] Danhier F., Ansorena E., Silva J.M., Coco R., Le Breton A., Préat V. (2012). PLGA-based nanoparticles: An overview of biomedical applications. J. Control. Release.

[30] Leonard F., Ali H., Collnot E.M., Crielaard B.J., Lammers T., Storm G., Lehr C.M. (2012). Screening of budesonide nanoformulations for treatment of inflammatory bowel disease in an inflamed 3D cell-culture model. ALTEX.

[31] Pereira A., Brito G., Lima M., Silva Júnior A., Silva E., de Rezende A., Bortolin R., Galvan M., Pirih F., Araújo Júnior R. (2018). Metformin Hydrochloride-Loaded PLGA Nanoparticle in Periodontal Disease Experimental Model Using Diabetic Rats. Int. J. Mol. Sci..

[32] Ensign L.M., Cone R., Hanes J. (2012). Oral drug delivery with polymeric nanoparticles: The gastrointestinal mucus barriers. Adv. Drug Deliv. Rev..

[33] Jain J.P., Kumar N. (2010). Development of amphotericin B loaded polymersomes based on (PEG)3-PLA co-polymers: Factors affecting size and in vitro evaluation. Eur. J. Pharm. Sci..

[34] Dos Santos-Silva A.M., De Caland L.B., de SL Oliveira A.L.C., De Araújo-Júnior R.F., Fernandes-Pedrosa M.F., Cornélio A.M., Da Silva-Júnior A.A. (2017). Designing structural features of novel benznidazole-loaded cationic nanoparticles for inducing slow drug release and improvement of biological efficacy. Mater. Sci. Eng. C.

[35] dos Santos Silva A.M., de Caland L.B., de Melo Doro P.N., de Sá Leitão Oliveira A.L.C., de Araújo-Júnior R.F., Fernandes-Pedrosa M.F., do Egito E.S.T., da Silva-Junior A.A. (2019). Hydrophilic and hydrophobic polymeric benznidazole-loaded nanoparticles: Physicochemical properties and in vitro antitumor efficacy. J. Drug Deliv. Sci. Technol..

[36] Leitão R.F.C., Ribeiro R.A., Bellaguarda E.A.L., MacEdo F.D.B., Silva L.R., Oriá R.B., Vale M.L., Cunha F.Q., Brito G.A.C. (2006). Role of nitric oxide on pathogenesis of 5-fluorouracil induced experimental oral mucositis in hamster. Cancer Chemother. Pharmacol..

[37] Sonis S.T., Tracey C., Shklar G., Jenson J., Florine D. (1990). An animal model for mucositis induced by cancer chemotherapy. Oral Surg. Oral Med. Oral Pathol..

[38] Ahmad N., Bhatnagar S., Saxena R., Iqbal D., Ghosh A.K., Dutta R. (2017). Biosynthesis and characterization of gold nanoparticles: Kinetics, in vitro and in vivo study. Mater. Sci. Eng. C.

[39] Sonis S.T., Peterson R.L., Edwards L.J., Lucey C.A., Wang L., Mason L., Login G., Ymamkawa M., Moses G., Bouchard P. (2000). Oral Oncol..

[40] De Araujo A.A., Varela H., De Medeiros C.A.C.X., De Castro Brito G.A., De Lima K.C., De Moura L.M., De Araujo R.F. (2015). Azilsartan Reduced TNF-α and IL-1β Levels, Increased IL-10 Levels and Upregulated VEGF, FGF, KGF, and TGF-α in an Oral Mucositis Model. PLoS ONE.

[41] Vilar C.J.F., Ribeiro S.B., de Araujo A.A., Guerra G.C.B., de Araujo Junior R.F., Brito G.A.D., Leitao R.F.C., Pontes D.L., Gasparotto L., Oliveira M.M.B. (2020). Effect of Gold Nanoparticle on 5-Fluorouracil-Induced Experimental Oral Mucositis in Hamsters. Pharmaceutics.

[42] Kendall C., Ionescu-Matiu I., Dreesman G.R. (1983). Utilization of the biotin/avidin system to amplify the sensitivity of the enzyme-linked immunosorbent assay (ELISA). J. Immunol. Methods.

[43] Rao X., Huang X., Zhou Z., Lin X. (2013). An improvement of the 2ˆ (–delta delta CT) method for quantitative real-time polymerase chain reaction data analysis. Biostat. Bioinform. Biomath..

[44] Singh V., Brecik M., Mukherjee R., Evans J.C., Svetlíková Z., Blaško J., Surade S., Blackburn J., Warner D.F., Mikušová K. (2015). The Complex Mechanism of Antimycobacterial Action of 5-Fluorouracil. Chem. Biol..

[45] Poetker D.M., Reh D.D. (2010). A Comprehensive Review of the Adverse Effects of Systemic Corticosteroids. Otolaryngol. Clin. N. Am..

[46] Fratoddi I., Venditti I., Cametti C., Palocci C., Chronopoulou L., Marino M., Acconcia F., Russo M.V. (2012). Functional polymeric nanoparticles for dexamethasone loading and release. Colloids Surf. B Biointerfaces.

[47] Gaudana R., Parenky A., Vaishya R., Samanta S.K., Mitra A.K. (2011). Development and characterization of nanoparticulate formulation of a water soluble prodrug of dexamethasone by HIP complexation. J. Microencapsul..

[48] Badran M.M., Alomrani A.H., Harisa G.I., Ashour A.E., Kumar A., Yassin A.E.B. (2018). Novel docetaxel chitosan-coated PLGA/PCL nanoparticles with magnified cytotoxicity and bioavailability. Biomed. Pharmacother..

[49] Feiner-Gracia N., Dols-Perez A., Royo M., Solans C., Garcia-Celma M., Fornaguera C. (2018). Cell penetrating peptide grafting of PLGA nanoparticles to enhance cell uptake. Eur. Polym. J..

[50] Ali H., Kalashnikova I., White M.A., Sherman M., Rytting E. (2013). Preparation, characterization, and transport of dexamethasone-loaded polymeric nanoparticles across a human placental in vitro model. Int. J. Pharm..

[51] Kunii R., Onishi H., Machida Y. (2007). Preparation and antitumor characteristics of PLA/(PEG-PPG-PEG) nanoparticles loaded with camptothecin. Eur. J. Pharm. Biopharm..

[52] Chronopoulou L., Domenici F., Giantulli S., Brasili F., D’Errico C., Tsaouli G., Tortorella E., Bordi F., Morrone S., Palocci C. (2019). PLGA based particles as “drug reservoir” for antitumor drug delivery: Characterization and cytotoxicity studies. Colloids Surfaces B Biointerfaces.

[53] Némethová V., Buliaková B., Mazancová P., Bábelová A., Šelc M., Moravčíková D., Kleščíková L., Ursínyová M., Gábelová A., Rázga F. (2017). Intracellular uptake of magnetite nanoparticles: A focus on physico-chemical characterization and interpretation of in vitro data. Mater. Sci. Eng. C.

[54] Assali M., Shawahna R., Shareef M., Alhimony I.-A. (2018). Dexamethasone-diclofenac loaded polylactide nanoparticles: Preparation, release and anti-inflammatory activity. Eur. J. Pharm. Sci..

[55] Gómez-Gaete C., Tsapis N., Besnard M., Bochot A., Fattal E. (2007). Encapsulation of dexamethasone into biodegradable polymeric nanoparticles. Int. J. Pharm..

[56] Doquet V., Barkia B. (2016). Combined AFM, SEM and crystal plasticity analysis of grain boundary sliding in titanium at room temperature. Mech. Mater..

[57] Erdagi S.I., Yildiz U. (2019). Diosgenin-conjugated PCL–MPEG polymeric nanoparticles for the co-delivery of anticancer drugs: Design, optimization, in vitro drug release and evaluation of anticancer activity. New J. Chem..

[58] Park J., Choi Y., Chang H., Um W., Ryu J.H., Kwon I.C. (2019). Alliance with EPR Effect: Combined Strategies to Improve the EPR Effect in the Tumor Microenvironment. Theranostics.

[59] Das S., Ongusaha P.P., Yang Y.S., Park J.-M., Aaronson S.A., Lee S.W. (2006). Discoidin Domain Receptor 1 Receptor Tyrosine Kinase Induces Cyclooxygenase-2 and Promotes Chemoresistance through Nuclear Factor-κB Pathway Activation. Cancer Res..

[60] Freudlsperger C., Bian Y., Wise S.C., Burnett J., Coupar J., Yang X., Chen Z., Van Waes C. (2013). TGF-β and NF-κB signal pathway cross-talk is mediated through TAK1 and SMAD7 in a subset of head and neck cancers. Oncogene.

[61] Liu J., Zhang M., Niu C., Luo Z., Dai J., Wang L., Liu E., Fu Z. (2013). Dexamethasone Inhibits Repair of Human Airway Epithelial Cells Mediated by Glucocorticoid-Induced Leucine Zipper (GILZ). PLoS ONE.

[62] Chinen J., Shearer W.T. (2010). Secondary immunodeficiencies, including HIV infection. J. Allergy Clin. Immunol..

[63] Vandevyver S., Dejager L., Van Bogaert T., Kleyman A., Liu Y., Tuckermann J., Libert C. (2012). Glucocorticoid receptor dimerization induces MKP1 to protect against TNF-induced inflammation. J. Clin. Investig..

[64] Chang W., Feng M., Li Y., Sun Y., Sun L. (2019). MKP1 overexpression reduces TNF-α-induced cardiac injury via suppressing mitochondrial fragmentation and inhibiting the JNK–MIEF1 pathways. J. Cell. Physiol..

[65] Shankar A., Roy S., Bhandari M., Rath G.K., Biswas A.S., Kanodia R., Adhikari N., Sachan R. (2017). Current Trends in Management of Oral Mucositis in Cancer Treatment. Asian Pac. J. Cancer Prev..

[66] Mak T.W., Yeh W.-C. (2002). Signaling for survival and apoptosis in the immune system. Arthritis Res..

